# Polymorphism discovery and allele frequency estimation using high-throughput DNA sequencing of target-enriched pooled DNA samples

**DOI:** 10.1186/1471-2164-13-16

**Published:** 2012-01-11

**Authors:** Michael P Mullen, Christopher J Creevey, Donagh P Berry, Matt S McCabe, David A Magee, Dawn J Howard, Aideen P Killeen, Stephen D Park, Paul A McGettigan, Matt C Lucy, David E MacHugh, Sinead M Waters

**Affiliations:** 1 Animal and Bioscience Research Department, Animal and Grassland Research and Innovation Centre, Teagasc, Athenry, Galway, Ireland; 2 Grange, Dunsany, Meath, Ireland; 3 Moorepark, Fermoy, Cork, Ireland; 4 Animal Genomics Laboratory, UCD School of Agriculture and Food Science, University College Dublin, Belfield, Dublin 4, Ireland; 5 Department of Animal Sciences, University of Missouri, Columbia, USA; 6 UCD Conway Institute of Biomolecular and Biomedical Research, University College Dublin, Belfield, Dublin 4, Ireland

## Abstract

**Background:**

The central role of the somatotrophic axis in animal post-natal growth, development and fertility is well established. Therefore, the identification of genetic variants affecting quantitative traits within this axis is an attractive goal. However, large sample numbers are a pre-requisite for the identification of genetic variants underlying complex traits and although technologies are improving rapidly, high-throughput sequencing of large numbers of complete individual genomes remains prohibitively expensive. Therefore using a pooled DNA approach coupled with target enrichment and high-throughput sequencing, the aim of this study was to identify polymorphisms and estimate allele frequency differences across 83 candidate genes of the somatotrophic axis, in 150 Holstein-Friesian dairy bulls divided into two groups divergent for genetic merit for fertility.

**Results:**

In total, 4,135 SNPs and 893 indels were identified during the resequencing of the 83 candidate genes. Nineteen percent (*n *= 952) of variants were located within 5' and 3' UTRs. Seventy-two percent (*n *= 3,612) were intronic and 9% (*n *= 464) were exonic, including 65 indels and 236 SNPs resulting in non-synonymous substitutions (NSS). Significant (*P *< 0.01) mean allele frequency differentials between the low and high fertility groups were observed for 720 SNPs (58 NSS). Allele frequencies for 43 of the SNPs were also determined by genotyping the 150 individual animals (Sequenom^® ^MassARRAY). No significant differences (*P *> 0.1) were observed between the two methods for any of the 43 SNPs across both pools (i.e., 86 tests in total).

**Conclusions:**

The results of the current study support previous findings of the use of DNA sample pooling and high-throughput sequencing as a viable strategy for polymorphism discovery and allele frequency estimation. Using this approach we have characterised the genetic variation within genes of the somatotrophic axis and related pathways, central to mammalian post-natal growth and development and subsequent lactogenesis and fertility. We have identified a large number of variants segregating at significantly different frequencies between cattle groups divergent for calving interval plausibly harbouring causative variants contributing to heritable variation. To our knowledge, this is the first report describing sequencing of targeted genomic regions in any livestock species using groups with divergent phenotypes for an economically important trait.

## Background

The somatotrophic axis (GH/IGF-1) is well established as central to nutrient partitioning, post-natal growth and development in mammals [1]. In domestic ruminants the influence of this axis on traits of commercial importance such as body size, carcass weight, milk yield and fertility has been widely published [2,3]. Genomic variation in key genes of the somatotrophic, such as insulin-like growth factor 1 (*IGF1*), growth hormone (*GH1*) and growth hormone receptor (*GHR*) have previously been shown to associate with production traits in dairy cattle [4-7]. Quantitative trait loci (QTL) that encompass *GHR *on BTA20 and *IGF-1 *on BTA5 associated with fertility traits have also been reported [8-11]. However, with the exception of *F279Y*, a non-synonymous SNP in exon 8 of *GHR *[8] strongly associated with milk yield and composition in dairy cattle, there is a dearth of information on candidate causal polymorphisms affecting performance in genes of this axis and its regulators.

In recent years, array-based genome wide association studies (GWAS) have improved our understanding of the genetic basis of complex traits in humans and other mammalian species [12-14]. However, a large proportion of the genetic variation underpinning complex traits cannot be explained using current GWAS approaches [15]. The contribution of variants segregating at very low frequencies, less than 0.05, termed rare variants, are thought to contribute to this 'missing heritability' and have typically been outside the scope of many GWAS array designs [15,16]. Recently proposed novel methods for haplotype analysis of high density arrays have demonstrated the ability, however, to identify genomic regions harbouring rare recessive variants affecting fertility in cattle [17]. The identification of putative genetic variants, including rare variants, underlying complex traits requires the analysis of large numbers of individual samples [18] and even with the rapid development of high-throughput sequencing technology and associated decreasing costs [19], sequencing of large numbers of individual genomes remains prohibitively expensive. While the development of custom targeted genome enrichment prior to sequencing is enabling analysis of large genomic regions of multiple genomes at reduced costs [20-22], the preparation of individual genomes for enrichment and sequencing is labour intensive and still beyond the capabilities of many research groups. Consequently, the pooling of DNA from subsets of samples prior to high-throughout sequencing to reduce sequencing costs is a viable alternative and has been successfully used to identify variants associated with complex traits in humans [23,24].

The aims of this study were to (1) identify putative coding and regulatory DNA sequence polymorphisms in 83 candidate genes of the somatotrophic axis, and (2) estimate allele frequency differences at these loci between pooled groups of dairy cattle divergent in genetic merit for fertility, using a pooled DNA approach coupled with 'sequence capture target enrichment' and high-throughput next generation sequencing technology. Estimated allele frequencies of a selection of SNPs from the sequence capture target enrichment and sequencing of pooled samples were compared to actual allele frequencies generated using Sequenom^® ^MassARRAY iPLEX™ gold assay. Results from this study will examine the pooled sequencing approach as an initial step for the identification of candidate genetic markers for fertility in dairy cattle and other complex performance traits in livestock.

## Methods

### Gene selection

A total of 83 genes were selected for targeted re-sequencing based on: (1) a comprehensive literature review of the somatotrophic axis, including its transcriptional regulators, binding proteins and associated genes involved in gluconeogenesis and insulin nutrient partitioning-related pathways; and (2) the availability of the DNA sequences in the Ensembl and/or GenBank databases (Additional File 1, Table S1).

### Animal Selection

Genomic DNA was available for 698 Holstein-Friesian progeny-tested artificial insemination (AI) bulls. An iterative algorithm was invoked to chose 150 bulls divided into two groups (n = 75) divergent for genetic merit (i.e. predicted transmitting ability) for calving interval while cognizant of the co-ancestry within each group. Firstly both genetic merit for calving interval and pairwise relationships among all animals were standardized to have equal variance. An index was derived for each animal using the (standardized) estimated breeding value of the animal and the (standardized) relationship of the animal to each of the other animals. The weighting on EBV and relatedness in the index was 60:40. An algorithm was subsequently invoked to generate the groups. Firstly, the animal with the lowest genetic merit for calving interval was selected and allocated to the low calving interval group. A second animal was selected based on its average index value with the first animal selected. Subsequently a third animal was selected based on its average index value with the bulls previously selected. This algorithm continued until 75 animals bulls were selected for the low calving interval group. The algorithm was again invoked to select the group of animals of high genetic merit for calving interval; the selection choice did not include any animal in the poor genetic merit group. This resulted in the sires in the high CIV group representing 46 different paternal half-sib groups and 44 different maternal grand-sires lines while the sires in the low CIV group represented 71 different paternal half sib groups and 61 different maternal grandsire lines. In total, 116 different sire lines (84 different paternal grand-sire lines) and 102 different maternal-grandsire lines were represented. The co-ancestry among the high CIV group was 3.0%, among the low CIV group was 0.24% and between the high and low CIV groups was 0.20%; the low CIV animals were on average 9 years older than the high CIV group. The median (inter-quartile range in parenthesis) number of daughters per sire and reliability were 160 (261) and 83% (23%) for the high CIV pool and 261 (738) and 79% (43%) for the low CIV pool. Mean predicted transmitting ability (standard deviation in parenthesis) for the 75 high and 75 low calving interval bulls was 5.3 days (1.6) and -5.8 days (1.4), respectively.

### Sample preparation, target enrichment and sequencing

For both sample groups (*n *= 75), DNA was pooled using equimolar quantities (100 ng) of DNA from each individual animal. The pools were then prepared for high-throughput DNA sequencing using the Illumina Genome Analyzer II platform. For this, 5 μg of pooled genomic DNA was sheared for 30 min using NEBNext^® ^dsDNA Fragmentase™ (New England Biolabs UK Ltd., Hitchin, UK) according to manufacturer's instructions. Blunt-end fragment repair and A tailing was performed on the resulting fragments using NEBNext^® ^End Repair Module and NEBNext^® ^dA-Tailing Module (New England Biolabs UK Ltd., Hitchin, UK). Illumina standard paired end (PE), adapters (Illumina, Essex, UK), including a 6 bp index, were ligated to the fragments, and the indexed adapter ligated fragments were gel purified and enriched by 12 cycles of PCR using Illumina PE1 and PE2 primers and Phusion High-Fidelity DNA Polymerase (New England Biolabs UK Ltd., Hitchin, UK).

Indexed PE sequencing libraries were captured and enriched for the genes of interest using the SureSelect Target Enrichment for Illumina^® ^PE Sequencing (Agilent Technologies Ltd., Cork, Ireland) according to manufacturer's instructions. Bovine Hybloc (Applied Genetics Laboratories, Florida, USA) was used instead of human Cot1 DNA during the sequence capture process to prevent non-specific hybridisation to the sequence capture baits. Sequence capture baits were designed to target whole gene (exons and introns) sequences including 3 kb of both the 5' and 3' flanking UTR sequence for 22 genes central to the function of the somatotrophic axis (Additional File 1, Table S1). To maximise the number of genes included for analysis, the remaining baits were designed to target only the coding sequences and 5' and 3' flanking UTR regions and encompassed 61 additional genes (Additional File 1, Table S1). In total, approximately 1.5 Mb of DNA sequence was targeted for capture. Target captured libraries from both sample groups contained different indexes located at the 5' end of both reads, allowing them to be pooled into a single flow cell lane. 80 bp PE sequencing was conducted on an Illumina GAIIx (cluster kit 4PE and sequencing kit version 5) and indexed sequencing reads from the two groups of animals were separated bioinformatically.

### Mapping and variant calling

All DNA sequence data were aligned to the *B. taurus *version 4.0 (Btau_4.0) reference genome using the Burrows-Wheeler Aligner (BWA) program version 0.5.9 [25] and the alignments were sorted and filtered for possible PCR and optical duplicates using Samtools version 0.1.17 [26]. The Genome Analysis Toolkit (GATK) version 1.0.5506 [27] was used for base quality score recalibration incorporating known Bovine SNPs from ENSEMBL [28] and indel realignment using standard hard filtering parameters [29]. DNA sequence polymorphisms were then identified for each of the sequenced regions using Samtools version 0.1.17 [26]. Samtools was also used with in-house scripts to calculate coverage estimates and to compare frequencies between the groups. Non-synonymous SNPs were identified using the Btau_4.0 annotation from ENSEMBL version 62 [30] using SNPdat (available upon request from the authors).

For variant calling, reads below stringent thresholds for mapping quality score (≤ 50) and base quality (≤ 20) were discarded. In addition, to call a variant a minimum of 4 reads supporting the non reference allele was required across both pools.

### Accuracy of SNP detection and allele frequency estimation

To assess the accuracy of SNP detection and allele frequency estimation we compared the high throughput DNA sequence data to: (1) SNPs located within the 1.5 Mb of targeted sequences as reported in the dbSNP (v130) database; and (2), SNPs validated, as part of a previous larger study performed by this group, as segregating in these 150 Holstein Friesian (HF) cattle using capillary based Sanger re-sequencing and Sequenom^® ^MassARRAY genotyping technologies. This included analysis of genotypes previously reported by this group in *IGF1 *[4], *IGF2R, IGF2 *[31,32], *GH1 *[5] and *GHR *[7].

### Transcription factor and microRNA binding site analysis

Bioinformatic analysis was performed on SNPs in the regulatory regions of selected genes to examine the effects of allele substitution on predicted transcription factor binding sites using MatInspector software package [33] and microRNA (miRNA) binding sites using MicroInspector software [34].

### Comparison of SNP allele frequency estimations

A two-tailed Fisher's exact test was used to 1) compare the allele frequency estimates generated using high throughput sequencing and Sequenom^® ^MassARRAY genotyping technologies for the SNPs in common across both platforms, and 2) compare the allele frequencies generated using high throughput sequencing in the pools of animals divergent for genetic merit for calving interval. In both analyses adjustment for multiple testing was undertaken using the Benjamini and Hochberg [35] correction for an experiment-wise significance of *P *< 0.1 and < 0.01 in the first and second analyses, respectively.

## Results

### High throughput DNA sequence analysis

**Figure 1:**
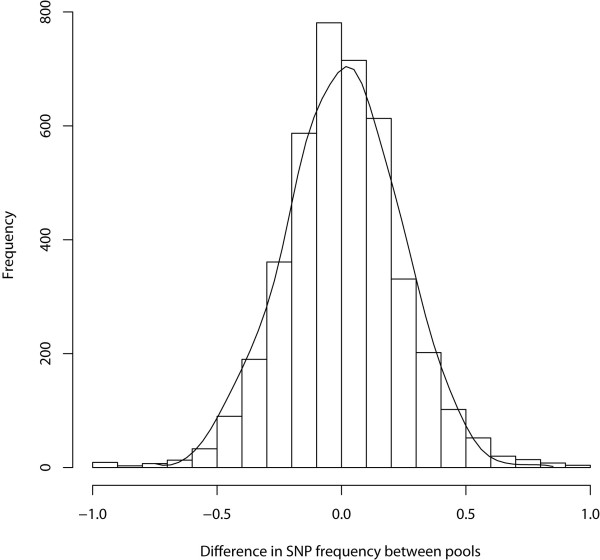
**Histogram of the distribution of the allele frequency differentials observed between high and low calving interval (CIV) pools**.

**Figure 2:**
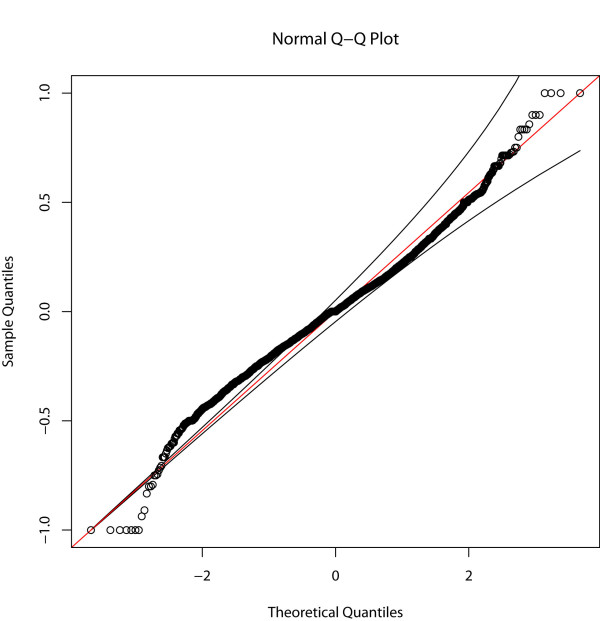
**Q-Q plot representing the distribution of the allele frequency differentials observed between high and low calving interval (CIV) pools**.

Approximately 2.95 million reads were obtained generating, on average, 1.05 Gb of sequence data per pool. Of the approximate 1.5 Mb of sequence targeted for enrichment, 1.2 Mb was sequenced with an average of 44-fold coverage per base across both pools (Table 1). We identified a total of 5,028 variants (4,135 SNPs and 893 indels) across the 83 genes (Additional File 1, Table S2). Nineteen percent (*n *= 952) of variants were located within 5' and 3' UTRs, 72% (*n *= 3,612) were intronic and 9% (*n *= 464) were exonic, including 65 indels and 236 SNPs resulting in non-synonymous substitutions [NSS] (Table 2). Significant (*P *< 0.01) allele frequency differentials between low and high CIV groups were observed for 720 SNPs including 58 NSS (Additional File 1, Table S2). The top 20 most significant SNPs differentiating the high and low CIV groups, located at least 10 Mb apart, in exonic, intronic, and regulatory regions is displayed in Table 3. Distribution of the 4, 135 SNP allele frequency differentials between both pools showed a slightly heavy tailed normal distribution (Figures 1 and 2).

**Table 1:** Capture efficiency

	Low calving interval pool	High calving interval pool
Total sequencing data generated (Gb)	1.57	1.38
Sequence data mapped to bovine genome (Gb)	1.23 (78%)	0.9 (67%)
Data mapped to targeted regions (Mb)	143 (12%)	132 (14%)
Data remaining after quality control (Mb)	74 (52%)	48 (34%)
Number of bases targeted (Mb)	1.54	1.54
Quantity of bases covered (Mb)	1.18 (76%)	1.20 (77%)
Average fold coverage per base	56	32

Percentages in parentheses represent proportions related to the category above each field.

**Table 2:** Variants detected across the 1.5Mb of targeted sequence

	Shared between pools		Unique in Low CIV pool^2^		Unique to High CIV pool^2^	
	
	SNP	Indel	SNP	Indel	SNP	Indel
5' and 3' UTR	547	86	93	50	118	58
Exonic^1^	278 (153)	29	57 (38)	17	65 (45)	19
Intronic	2226	277	310	189	441	168

^1^: Numbers in parenthesis represent the number of SNPs resulting in non synonymous substitutions; ^2^: Variants classified as unique represent variants undetected in the corresponding pool.

**Table 3:** Top 20 most significant SNPs, at least 10 Mb apart, in exonic, intronic and UTR regions displaying differential frequencies between groups of cattle divergent for calving interval.

Entrez gene/Ensembl ID	**Chr**.	Location	Allele	Frequency in Low CIV pool^1^	Frequency in High CIV pool^1^	*P value^2^*	Type/Location^3^	Predicted effect^4^	Reference^5^
*GH1*	19	49749042	G>T	0.00	0.38	*9.2 *× *10^-15^*	Exon NSS	N/A	rs41917096
*SST*	21	49258728	C>T	0.00	0.34	4.8 × *10^-9^*	Exon NSS	N/A	novel
*NR2F2*	21	9510000	A>G	0.00	0.52	9.1 × *10^-8^*	Exon NSS	N/A	novel
*HK3*	7	37630361	T>G	0.00	0.30	8.2 × *10^-6^*	Exon NSS	N/A	novel
*STAT5B*	19	43679543	G>T	0.00	0.28	1.2 × *10^-5^*	Exon NSS	N/A	novel
*IGFBP5*	2	108855684	C>T	0.00	0.54	2.3 × *10^-12^*	5'	2 × pTFBS	novel
*MAPK9*	7	871347	C>T	0.47	0.09	5.3 × *10^-12^*	5'	1 × pTFBS	rs43495395
*GCK*	11	74275999	G>A	0.00	0.39	2.2 × *10^-9^*	5'	8 × pTFBS	novel
*GHR*	20	34207771	C>A	0.00	0.39	8.3 × *10^-9^*	5'	None	novel
*HK1*	28	24994609	C>T	0.00	0.18	2.4 × *10^-7^*	3'	1 × miRNA	novel
*GHRH*	13	66803046	A>G	0.65	0.25	3.1 × *10^-6^*	5'	None	novel
*IRS4*	X	35195377	C>G	0.00	0.83	4.9 × *10^-9^*	3'	None	novel
*SLC2A1*	3	110250920	T>G	0.00	0.38	5.0 × *10^-9^*	3'	4 × miRNA	novel
*SLC5A1*	17	73990477	C>T	0.68	0.18	9.7 × *10^-7^*	3'	None	rs41255339
*ESR2*	10	78593544	C>T	0.00	0.21	9.3 × *10^-7^*	3'	1 × miRNA	novel
*IGF2R*	9	100136966	C>T	0.79	0.00	5.5 × *10^-20^*	Intron	N/A	novel
*PIK3R2*	7	4996360	C>A	0.00	0.55	2.8 × *10^-15^*	Intron	N/A	novel
*Q95M43*	7	14524938	G>A	0.00	0.44	4.4 × *10^-9^*	Intron	N/A	novel
*IGFBP3*	4	78896406	A>T	0.00	0.42	8.8 × *10^-9^*	Intron	N/A	novel
*SIRT2*	18	48205429	T>A	0.00	0.53	1.6 × *10^-8^*	Intron	N/A	novel

^1^: Frequency of second allele displayed; ^2^: Benjamini and Hochberg corrected *P *value; ^3^: NSS = SNPs resulting in non synonymous substitutions; ^4^: Predicted effects on transcription factor binding sites in the 5' regulatory regions using MatInspector software package [33] and on microRNA binding sites in the 3' regulatory regions using MicroInspector software [34], detailed information on predicted effects displayed in Tables 6 and 7; ^5^: SNPs classified according to dbSNP (http://www.ncbi.nlm.nih.gov/projects/SNP/ accessed 9^th ^September 2011).

### Accuracy of SNP detection and allele frequency estimation

#### Comparison to dbSNP

**Figure 3:**
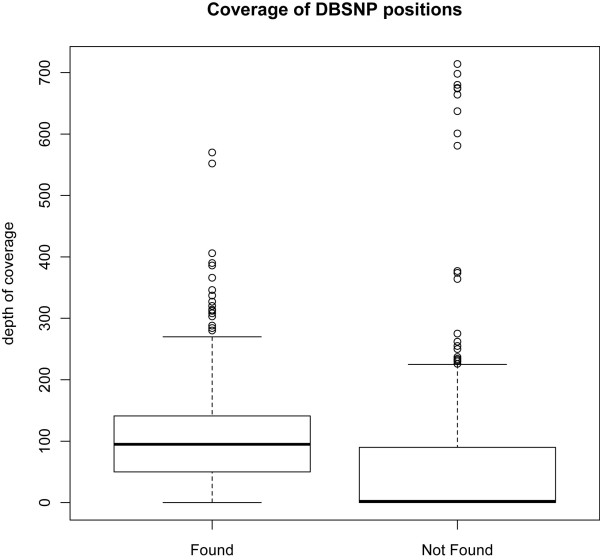
**Box plot representing the quartile distribution of sequencing depth in the detected and undetected SNP groups compared to dbSNP v130**.

In total, 1,304 SNPs were reported in dbSNP v130 across the 1.5 Mb of targeted sequences. Of these, 598 SNPs were identified during the high throughput sequencing with 706 SNPs undetected (Additional File 1, Table S3). A large number of undetected SNP loci had less than 4× coverage (*n *= 396) in the present study. Assuming all SNPs reported in dbSNP were present in this population of HF sires, the false negative rate drops to 28.5% (268 SNPs) and 26% (252 SNPs) when considering bases with at least 10× and 30× coverage, respectively. The median for base coverage in undetected and detected groups was two and 95 respectively (Figure 3). Comparison of these data with SNPs reported in dbSNP (v130) also identified 3537 putative novel SNPs (Additional File 1, Table S2).

#### Comparison to previous data from this group

**Figure 4:**
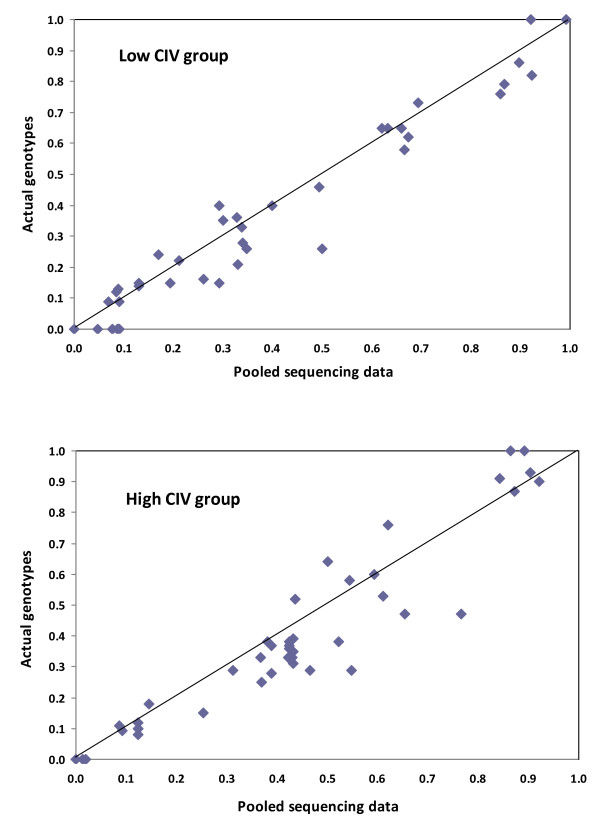
**Comparison of allele frequencies^1 ^estimated using high-throughput sequencing vs. genotyping for 43 SNPs across two pools of 75 dairy cattle divergent for calving interval (CIV)**. ^1^Actual genotype frequencies calculated from Sequenom^® ^MassARRAY data obtained from previous studies [4,5,7,31,32].

Analysis of the previous studies on these sires [4,5,7,31,32] identified 67 validated SNPs segregating in these 150 HF cattle, of which, 43 SNPs were identified in at least one of the two pools in this study (Table 4). The lowest minor allele frequency detected was 0.08 and 0.09 in the low and high CIV pools, respectively (Table 4). There was strong concordance between both methods with no significant differences (experiment-wise *P *> 0.1) between allele frequency estimates for any of the 43 SNPs across both low and high CIV pools (Table 4; Figure 4). The 24 undetected SNPs included 16 SNP loci with zero coverage, two SNP loci with combined coverage across both pools of less than 4× and six SNP loci with coverage depth of between 5× to 54× with variant allele frequencies ranging between 0.01 to 0.89 (Additional File 1, Table S4). The false negative rate reduced to 7.5% (5/67) and 3.0% (2/67) when considering variant loci with at least 10× and 30× coverage, respectively. Analysis of the allele frequencies for the two undetected SNPs with greater than 30× coverage revealed they were below detectable thresholds given their respective coverage at each loci (Additional File 1, Table S4).

**Table 4:** Comparison between allele frequencies generated using Sequenom^® ^MassARRAY and Illumina GAIIx technologies.

EntrezGene ID	**Chr**.	SNP name^1^	Position	Allele sub.^2^	Allele frequency in low CIV pool^3^		*P *value^4^	Allele frequency in high CIV pool^3^		*P *value^4^	Reference^5^
					**Actual**	**NGS**		**Actual**	**NGS**		
		*S555G*	33897071	T > C	0.19	0.15	1.00	0.12	0.10	1.00	AF140284
		*H545*	33897099	A > G	0.34	0.28	0.96	0.15	0.18	1.00	AF140284
		*A536T*	33897128	C > T	0.13	0.15	1.00	0.02	0.00	1.00	AF140284
		*N528T*	33897151	T > G	0.21	0.22	1.00	0.12	0.10	1.00	AM161140
		*GHR76*	33897252	A > G	0.13	0.14	1.00	0.02	0.00	1.00	n/a
		*F279Y*	33915503	A > T	0.00	0.00	1.00	0.09	0.09	1.00	AM161140
		*GHR19.1*	33994639	G > T	0.62	0.65	1.00	0.47	0.29	0.23	rs109702942
		*GHR18.2*	33995251	G > C	0.07	0.09	1.00	0.00	0.00	1.00	n/a
		*AF126288:g.149*	34086084	C > T	0.69	0.73	1.00	0.31	0.29	1.00	AF126288
*GHR*	20	*GHR9.1*	34101240	C > T	0.05	0.00	0.96	0.37	0.33	1.00	rs110979028
		*GHR3.3*	34166627	A > G	0.08	0.12	1.00	0.43	0.39	1.00	n/a
		*GHR3.2*	34166731	A > G	0.09	0.13	1.00	0.43	0.31	0.96	n/a
		*GHR3.1*	34166898	T > C	0.09	0.00	0.23	0.42	0.33	0.96	n/a
		*GHR2.6*	34166944	C > T	0.09	0.00	0.22	0.42	0.38	1.00	rs109825954
		*GHR2.5*	34166970	A > G	0.09	0.00	0.23	0.42	0.36	1.00	n/a
		*GHR2.4*	34166982	A > C	0.09	0.00	0.22	0.43	0.33	0.96	n/a
		*GHR2.3*	34167025	C > T	0.09	0.00	0.23	0.42	0.37	1.00	n/a
		*GHR2.2*	34167126	G > A	0.09	0.09	1.00	0.43	0.35	1.00	n/a
		*GHR2.1*	34167240	C > T	0.09	0.00	0.13	0.44	0.52	1.00	n/a
	
		*GH19*	49657225	G > A	0.90	0.86	1.00	0.84	0.91	1.00	rs41923481
		*GH18*	49657293	G > A	0.92	0.82	1.00	0.92	0.90	1.00	rs196003433
		*GH17*	49657371	C > G	0.92	1.00	1.00	0.89	1.00	1.00	rs41923483
		*2291*	49660125	T > G	0.08	0.00	0.13	0.12	0.08	1.00	n/a
		*2141*	49660275	G > C	0.33	0.36	1.00	0.09	0.11	1.00	rs41923484
		*GH6*	49660469	A > C	0.08	0.00	0.96	0.12	0.12	1.00	rs196003424
		*GH38*	49693278	G > A	0.33	0.21	1.00	0.59	0.60	1.00	rs41923525
*GH1*	19	*GH37*	49693285	C > G	0.29	0.15	0.96	0.54	0.58	1.00	rs41923524
		*GH36*	49693316	G > C	0.29	0.40	1.00	0.52	0.38	1.00	rs41923523
		*GH35*	49693328	G > A	0.30	0.35	1.00	0.55	0.29	0.96	rs41923522
		*GH34*	49693374	C > G	0.34	0.33	1.00	0.61	0.53	1.00	rs41923521
		*GH32*	49693442	A > G	0.67	0.58	1.00	0.39	0.37	1.00	rs196301608
		*GH31*	49693460	C > T	0.67	0.62	1.00	0.39	0.28	1.00	rs196003442
		*GH30*	49693512	A > G	0.66	0.65	1.00	0.38	0.38	1.00	rs196003441
		*GH29*	49693686	C > T	0.50	0.26	0.36	0.50	0.64	0.96	rs41923520
	
		*rs29012855*	71150007	C > T	0.99	1.00	1.00	0.90	0.93	1.00	rs29012855
		*IGF1i3*	71175747	T > C	0.17	0.24	0.95	0.01	0.00	1.00	rs109557731
*IGF1*	5	*IGF1i2*	71175753	A > G	0.49	0.46	1.00	0.25	0.15	0.70	rs109227434
		*IGF1i1*	71176219	A > T	0.26	0.16	1.00	0.37	0.25	1.00	rs110076130
		*AF017143*	71198324	G > A	0.35	0.26	0.96	0.62	0.76	0.92	AF017143
	
*IGF2*	29	*IGF2_B_6646*	51250879	A > G	0.40	0.40	1.00	0.65	0.47	0.83	rs42196909
	
		*IGF2R_D_41515*	100112956	A > G	0.87	0.79	1.00	0.87	0.87	1.00	rs41623543
*IGF2R*	9	*IGF2R_D_41092*	100113379	G > A	0.63	0.65	1.00	0.77	0.47	0.22	rs41623544
		*IGF2R:g.86262*	100134604	G > A	0.86	0.76	0.73	0.86	1.00	0.62	n/a

^1^: SNP details according to previous studies [4,5,7,31,32]; ^2^: Reported from the forward strand; ^3^: Allele frequencies represents second allele; ^4^: Benjamini and Hochberg corrected *P *values; ^5^:Genbank accession or dbSNP reference (http://www.ncbi.nlm.nih.gov/projects/SNP/ accessed 9^th ^September 2011).

#### Transcription factor and microRNA binding site analysis

Analysis of three SNPs located in the 5' UTR of insulin-like growth factor binding protein 5 (*IGFBP5)*, the mitogen-activated protein kinase 9 gene (*MAPK9*) and the glucokinase gene (*GCK*) were predicted to collectively modulate 11 transcription factor binding sites (TFBS) (Table 5). The two SNPs analysed in the 5' UTR of *GHR *and growth hormone releasing hormone (*GHRH*) were not predicted to affect any TFBS.

**Table 5:** Effects of SNPs located in the 5' UTR of *IGFBP5*, *MAPK9 *and *GCK *on predicted transcription factor binding sites.

Entrez Gene ID	**Chr**.	Position	Allele	Strand	Matrix Family	Core similarity	Matrix similarity	Site sequence	Detailed Family Information
*IGFBP5*	2	108855684	T	(-)	V$ZF07	1.00	0.94	ggtccCTCCtctcag	C2H2 zinc finger transcription factors 7
				(+)	V$PLAG	1.00	0.89	gaGAGGagggacccaggggaggg	Pleomorphic adenoma gene
	
*MAPK9*	7	871347	C	(+)	V$NFKB	1.00	0.93	aacgggtgTTCCttc	Nuclear factor kappa B/c-rel
	
				(+)	V$EREF	1.00	0.81	cagggaggactgtgtGACCtggt	Estrogen response elements
				(-)	V$PPAR	0.76	0.71	aacCAGGtcacacagtcctccct	Peroxisome proliferator activated receptor homodimers
			G	(-)	V$PAX3	1.00	0.87	caggTCACacagtcctccc	PAX-3 binding sites
*GCK*	11	74275999		(-)	V$EREF	1.00	0.93	aaaccagGTCAcacagtcctccc	Estrogen response elements
				(-)	V$RORA	1.00	0.96	agaaaaccaGGTCacacagtcct	v-ERB and RAR-related orphan receptor alpha
			
				(+)	V$GREF	0.75	0.86	ggaggactgtgTGATctgg	Glucocorticoid responsive and related elements
			A	(-)	V$GATA	0.85	0.90	accaGATCacaca	GATA binding factors
				(-)	V$RXRF	1.00	0.79	agcaaaGGTCagaaaaccagatcac	RXR heterodimer binding sites

The "core sequence" of a matrix is defined as the (usually 4) consecutive highest conserved positions of the matrix (marked in uppercase letters). A perfect match between the consensus bovine sequence and the matrix gets a score of 1.00 (each sequence position corresponds to the highest conserved nucleotide at that position in the matrix); a "good" match to the matrix usually has a similarity of >0.80.

Analysis of the five SNPs located in the 3' UTR of Hexokinase 1 (*HK1*), carrier family 2 (sodium/glucose cotransporter) member 1 gene (*SLC2A1*), insulin receptor substrate 4 *(IRS4), estrogen receptor beta (ESR2) *and carrier family 5 (sodium/glucose cotransporter) member 1 gene (*SLC5A1*) predicted SNPs located in *HK1, SLC2A1 *and *ESR2 *affected binding of six microRNAs while the remaining two SNPs in *IRS4 *and *SLC5A1 *were not predicted to have any effects (Table 6).

**Table 6:** Effects of SNPs located in the 5' UTR of *SLC2A1*, *HK1 *and *ESR2 *on predicted miRNA binding sites.

Entrez Gene ID/Ensembl ID	Chromosome	Position	Allele	Strand	miRNA name	miRNA sequence	Free energy ΔG^1 ^(kcal/mol)
			T	(+)	bta-miR-10b	uacccuguagaaccgaauuugug	-20.7
			
*SLC2A1*	3	110250920			bta-miR-136	acuccauuuguuuugaugaugga	-22.0
			G	(+)	bta-miR-1249	acgcccuucccccccuucuuca	-21.5
					bta-miR-2284r	uuggcccaaaaguucguucggau	-21.3
			
*HK1*	28	24994609	T	(+)	bta-miR-2465	ugagccacaguagagccuuggau	-21.9
			
*ESR2*	10	78593544	A	(-)	bta-miR-2348	uucgggugguguggagcggcc	-21.9

^1^Free energy: Gibbs free energy (ΔG) of the duplex structure is indicated.

## Discussion

### DNA pooling and allele frequency estimation

DNA pooling and comparison of allele frequencies between groups of individuals divergent for a particular phenotype is an attractive approach to candidate QTN identification primarily due to the current costs of target enrichment and high throughput sequencing of large numbers of individual genomes [36]. Although segregation at significantly different frequencies between pools does not necessarily infer a relationship with the trait and may be a result of genetic drift or high linkage disequilibrium with a causative variant, this approach efficiently captures the genetic variation of individuals divergent for a particular phenotype and has been successfully used to identify variants involved in complex traits in humans [23,24]. However, the success of this approach is influenced by several factors including: (1) the degree of divergence of individuals for the true genetic merit of the trait as well as the effective number (i.e., after accounting for co ancestry) of individuals per pool; (2) equimolar pooling of DNA from each individual; (3) bias introduced during target enrichment prior to sequencing; (4) bias introduced during amplification during sequencing; (5) classification of variants during post sequencing data analysis; (6) sequencing error rate; (7) technical differences between sequencing lanes and (8) sampling bias during sequencing. Analysis of all the technical parameters individually was not within the remit of this study and has previously been discussed [22,36-39].

In the current study, we assessed performance of the process retrospectively by comparing the allele frequency estimates with results from conventional genotyping and observed a strong concordance between both methods even at low read depths of less than 10× where reliable sequencing data can be difficult to achieve [39]. Although the relative contribution of each sample in pooled sequencing is a critical issue and cannot be guaranteed, the high concordance with actual genotypes provided strong evidence that minimal biases were introduced including during in-solution enrichment which captured approximately 80% of the target sequence and has previously been reported to yield better uniformity and specificity than equivalent array based capture approaches [40]. Potential biases due to technical variations such as mechanical differences in sequencing lane manufacture [39] were circumvented by indexing groups and pooling into a single lane. However, despite sequencing within a single flow cell lane, differences in capture efficiencies were observed between pools. The high CIV pool generated 37% more data mapping to the bovine genome compared with the low CIV pool. Although, the authors cannot explain the differences, it is noteworthy that other authors have also observed differences in capture efficiencies between pooled DNA samples. For example, Bansal *et al*. (2011) [36] observed up to a 26% difference in sequencing coverage between libraries captured using the same target capture system. Furthermore, Maricic *et al*. 2010 [41] reported up to a four-fold difference in the number of sequence reads obtained using captured mitochondrial DNA sequences from 46 human individuals using a similar bait-design sequence capture system.

Despite the cost effective advantages a pooled sample approach delivers, given a fixed quantity of sequence data, a compromise on the fold-coverage per pooled sample/group and thereby sensitivity is unavoidable. The combined average read coverage of 88× across both pools impacted the sensitivity to detect variants segregating at low frequencies in either pool. Accounting for the requirement of 4 non-reference alleles across both pools to be present to call a variant translates to the ability to detect alleles with MAF, on average, of 4.5%. To achieve detection of alleles with MAF < 4.5% a reduction in the quantity of sequence targeted for enrichment and/or number of pools per sequencing lane would be required. This is an important consideration for study designs incorporating a DNA pooling and sequencing approach for rare variant detection. However a reduced ability to identify rare variants by sequencing many individuals at a more shallow depth in larger pool sizes can be offset by the gains in power achieved by more accurate estimation of allele frequencies compared to sequencing fewer individuals at higher depth with smaller pool size, even accounting for higher than expected error rates [42].

When assessing false negative rates in relation to reference databases other factors other than sequencing depth need consideration including segregation of these variants in the target population and accuracy of variants reported in the reference database. Poor sequencing depth was the main factor in the false negative rates found when compared to the Sequenom^® ^dataset as the majority of undetected SNP loci, i.e. 93% had low read depths of less than 10×. Comparison to the dbSNP database however highlighted that other factors were involved with only 61% of undetected SNPs having read depths less than 10×. The high SNP false negative rate of 20.5% (loci with >10× coverage) compared to dbSNP is most probably due to a combination of a lack of segregation of these SNPs in HF cattle and inaccurate dbSNP data. In support of this a recent commentary by Day (2010) [43] on the human dbSNP database revealed that several studies have reported discontinuity with dbSNP variants and depending on the study dbSNP false positive rates ranged between 8 - 17%.

### Identification of candidate causative variants

The identification of causative mutations or quantitative trait nucleotides (QTN) underlying performance traits in livestock is problematic with only a small number identified to date [44,45]. This is mainly due to the polygenic nature of quantitative traits requiring dense genome wide marker or sequence analysis on large populations of animals with accurate phenotypic data to identify and accurately estimate small effects especially on lowly heritable traits [14]. Other factors include the long generation interval of livestock, costs involved, lack of inbred lines, the difficulty of producing 'knock-out's [45] as well as possible conservation of LD within small chromosomal regions.

The somatotrophic axis is a likely candidate for harbouring QTN due to its central role in animal post-natal growth, development, lactogenesis, and reproduction [2,3]. It is therefore not surprising several groups have reported associations with variants in this axis and performance [6,8,46-51]. In addition to milk production and growth traits we have previously observed associations between calving interval and variants in *GHR *[7] and associations between an indirect measurement of reproductive performance (functional survival) and SNPs in both *GH1 *and *IGF1 *[4,5]. Our previous studies involved sequence analysis of specific regions, encompassing between only 2-5% of the sequence of each gene. Polymorphisms presented herein are the first genomic characterisation of this axis in cattle divergent for a performance trait, and were generated from sequencing entire genes and regulatory regions. It is therefore probable, even allowing for other possible genetic mechanisms such as copy number variation or epigenetic effects such as methylation, a subset of these variants underlies heritable variation in CIV. Although CIV is a lowly heritable trait (0.03-0.04; Berry *et al*. [52]) the sires used in the present study were of relatively high reliability. We identified variants (*n *= 301) within coding regions of 72 genes, consisting of either SNPs resulting in non-synonomous substitutions or indels, which could plausibly affect abundance or biological activity of their respective gene products. In this study, 58 of these SNPs were segregating at significantly different frequencies (*P *< 0.01) between the high and low CIV pools, all with at least 30× coverage, and warrant further investigation. In addition, SNPs in the regulatory regions flanking each gene were found to be present at different frequencies between pools (*n *= 116) and may harbour variants of biological significance. Interestingly, bioinformatic analysis of the top 10 most significant variants located in untranslated regions revealed SNPs located in the 5' UTR of *IGFBP5*, *MAPK9 *and *GCK *were predicted to collectively modulate 11 transcription factor binding sites (TFBS) and SNPs in the 3' UTR of *HK1, SLC2A1 *and *ESR2 *were predicted to modulate six miRNA binding sites. While in contrast significant SNPs analysed in the 5' UTR of *GHR *and *GHRH *and 3' region of *SIRT2 *and *SLC5A1 *were not predicted to have any effects on TF or miRNA binding. Perhaps not surprisingly, by far the largest proportion of all detected variants, 71% (*n *= 3612), were located in the intronic regions of the 22 genes targeted for complete sequencing, of which, frequencies of 524 SNPs were significantly different between groups. An example of the potential impact of intronic polymorphisms on gene function can be seen with one of the few QTNs identified in livestock, resulting in a major effect on muscle growth in pigs, is located within an intron of *IGF2 *[53]. While it is interesting to investigate possible effects of these polymorphisms, it is important to reiterate the observation of differential frequencies between pools does not translate to an association with CIV but instead candidate causative variants are presumably captured and cannot be identified until subsequent genotyping and association analysis.

Genotyping all identified variants across a large population of cattle with detailed phenotypic information would provide the greatest chance for QTN identification. However due to a combination of (1) the quantity of variants identified and (2) the requirement for large numbers of genotyped individuals to attain sufficient power in the association analysis renders this a costly approach. Therefore careful selection of candidate polymorphisms prior to genotyping will be required. A parameter worth consideration during variant selection is the likely extent of linkage disequilibrium (LD) between variants in either pool. High LD could result in substantial numbers of variants displaying differential frequencies due to nothing more than physical proximity to the causative agent. One limitation of the current DNA pooling strategy however is the inability to estimate LD and subsequent variant selection could inadvertently omit QTN candidates from genotyping. Selecting variants per gene/chromosome rather than genome wide and using bioinformatic tools to extrapolate possible biological effects based on our current understanding of gene regulation and function could reduce the number of false positives and negatives carried through the process. LD in cattle was previously thought to span large distances [54,55] but more recent evidence suggests the extent of LD in HF dairy cattle to be smaller in the region of 2 Mb (*r*^2 ^= 0.3) to 10 Mb (*D' *= 0.3) [56]. The current study identified 720 SNPs displaying significantly different allele frequencies between high and low CIV pools, located across 72 genes on 28 chromosomes with 50 of these genes separated by at least 10 Mb. Even considering the possibility of regions of high LD these results tentatively support previous observations of multiple independent effects between variants in genes of the somatotrophic axis and performance [57]. This is consistent with Fishers classical infinitesimal model of complex traits, where many genes are involved, each with small but additive effects [58].

This study is one of only two reporting the use of targeted enrichment for the analysis of large genomic regions in cattle, the previous study utilised high-throughput sequencing to identify the causative mutation underlying bovine arachnomelia, a congenial anomaly resulting in limb bone deformation [59]. To our knowledge, this report describes the first sequencing of targeted genomic regions using groups of individuals divergent for an economically important trait in livestock and the high concordance obtained between actual genotype frequencies and this data supports DNA pooling as a cost-effective alternative to individual animal genotyping for SNP allele frequency estimation in agreement with previous studies [36,38,60-63].

These results represent a preliminary screen for candidate causal polymorphisms in genes of the somatotrophic axis contributing to differences in genetic merit for CIV performance. Future work will include variant selection, aided by bioinformatic analysis, followed by genotyping on a large panel of cattle with detailed fertility information. As sequencing technology develops whole genome sequencing of large numbers individual genomes will become affordable for many study designs, but until then the detection of candidate causative variants, rare and common, via targeted re-sequencing followed by array based association studies will almost always be the most efficient design.

## Conclusion

This study validates the use of pooled DNA samples for subsequent enrichment and high-throughput sequencing as an accurate cost effective method to identify polymorphisms segregating at differential frequencies between populations and therefore may aid the identification of causative variants associated with complex traits.

## Authors' contributions

MPM contributed to study conception and design, preparation and pooling of DNA samples for target enrichment and sequencing, data analysis and drafted the manuscript. CJC performed the bioinformatic analysis of the sequencing data and contributed to the manuscript. MSM contributed to the study design, DNA pooling, preformed the target enrichment and prepared DNA libraries for sequencing. DPB contributed to study conception and design, collected the phenotypic data and selected the animals used in this study, performed statistical analyses and contributed to the preparation of the manuscript. DAM contributed to study design, DNA pooling, designed the baits for target enrichment and contributed to the manuscript. DJH extracted DNA from the semen samples used in this study. APK contributed to the DNA pooling. SDP and PAM contributed to the target enrichment bait design. MCL contributed in the selection of genes for sequencing. SMW and DEM participated in study conception, design and coordination and reviewed the manuscript. All authors read and approved the final manuscript.

## Supplementary Material

Additional file 1 **Excel file containing the four additional tables**. Additional Table 1 - details of the genes including sequence co ordinates selected for target enrichment and sequencing. Additional Table 2 - details of the SNPs and indels identified across the 83 candidate genes in both the low and high calving interval DNA pools. Additional Table 3 - details of all SNPs reported on dbSNPv130 within the 1.5 Mb DNA sequences targeted for sequencing. Additional Table 4 - 24 undetected SNPs validated as segregating in the 150 Holstein-Friesian sires using Sequenom^® ^MassARRAY genotyping. Click here for file
